# Impact of sex differences on clinical characteristics of anti-N-methyl-D-aspartate receptor encephalitis during the acute phase: a single-center retrospective study

**DOI:** 10.3389/fnhum.2025.1691391

**Published:** 2026-01-08

**Authors:** Xuan Zou, Guan-en Zhou

**Affiliations:** 1 Department of Neurology, Tianjin Huanhu Hospital, Tianjin University, Tianjin, China; 2 Clinical College of Neurology, Neurosurgery and Neurorehabilitation, Tianjin Medical University, Tianjin, China

**Keywords:** acute phase, anti-NMDAR encephalitis, autoimmune encephalitis, clinical characteristics, sex differences

## Abstract

**Objective:**

To elucidate the influence of sex differences on the clinical characteristics of anti-NMDAR encephalitis during the acute phase.

**Methods:**

Patients diagnosed with anti-NMDAR encephalitis who were hospitalized at Huanhu Hospital, affiliated with Tianjin University, from January 2020 to January 2025 were collected. They were divided into two groups: male and female. Clinical data for both groups were gathered, including age, history of prodromal infection, clinical manifestations, complications, presence of tumor, laboratory indices, MRI findings, GCS scores, length of hospital stay, treatment regimens, and acute phase outcomes. Statistical methods were employed to compare the differences between the two groups.

**Results:**

A total of 43 patients with anti-NMDAR encephalitis were included in this study, comprising 20 male patients (46.51%) and 23 female patients (53.49%). Female patients were more likely to exhibit decreased levels of consciousness compared to male patients (*χ^2^* = 4.113, *p* = 0.043). Additionally, the antibody titers in CSF of female patients were significantly higher than those in male patients too (*Z* = −2.870, *p* = 0.004). Interestingly, CSF protein levels were higher in male patients than in female patients (*Z* = −2.591, *p* = 0.019), and male patients were more prone to test positive for anti-MOG antibodies (*χ^2^* = 5.715, *p* = 0.017). The treatment improvement rate for female patients was lower than that for male patients (*Z* = 4.768, *p* = 0.029), and family members of female patients were more likely to automatic discharge (*χ^2^* = 4.075, *p* = 0.044).

**Conclusion:**

Female patients with anti-NMDAR encephalitis experience greater challenges and difficulties compared to male patients. Therefore, it is necessary to choose more proactive treatment options for female patients to help reduce the risk of adverse outcome in acute phase.

## 1 Introduction

Anti-NMDAR (anti-N-methyl-D-aspartate receptor) encephalitis is the most common type of autoimmune encephalitis (AE), which is also the first AE to be found with anti-neuronal cell surface antibodies (Dalmau et al., 2007). Anti-NMDAR encephalitis is closely associated with ovarian teratomas and primarily occurs in young women (Dalmau et al., 2011). Male patients with anti-NMDAR encephalitis are less common than female patients, and cases involving tumors are rare (Dalmau et al., 2008).

Sex factors influence the prevalence of anti-NMDAR encephalitis, but their effect on disease progression remains unclear. Currently, there is limited literature on this topic. This study aims to clarify the impact of sex differences on the clinical characteristics of anti-NMDAR encephalitis during acute phase, with the goal of guiding clinical diagnosis and treatment.

## 2 Materials and methods

### 2.1 Included samples

Patients with anti-NMDAR encephalitis who were hospitalized at Tianjin Huanhu Hospital, affiliated with Tianjin University, from January 2020 to January 2025 were included in the study. The patients usually experience five stages of clinical manifestations: the prodromal phase, psychotic and/or seizure phase, unresponsive and/or catatonic phase, hyperkinetic phase, and gradual recovery phase (Lin and Lin, 2020). In this study, the term “acute phase” is defined as all stages prior to stabilization of the illness and discharge, primarily encompassing the first four stages and the early recovery phase. Antibodies against the neuronal cell surface antigens in cerebrospinal fluid (CSF) and blood samples were detected using cell-based assay (CEA) and tissue-based assay (TBA) of indirect immunofluorescence assay (IIF). Additionally, immunoblotting was employed to identify antibodies against intracellular antigen of nerve cells.

**Figure 1:**
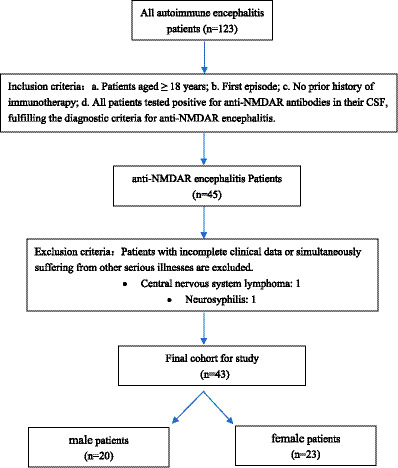
Flowchart of patient inclusion.

Inclusion criteria: a. Patients aged ≥ 18 years; b. First episode; c. No prior history of immunotherapy; d. All patients tested positive for anti-NMDAR antibodies in their CSF, fulfilling the diagnostic criteria for anti-NMDAR encephalitis (Graus et al., 2016). Exclusion criteria: patients with incomplete clinical data or simultaneously suffering from other serious illnesses are excluded. The enrolled patients were subsequently categorized into male and female groups. Flowchart of patient inclusion is shown in Figure 1.

### 2.2 Clinical data collection

Clinical data for both groups were collected, including patients’ age, history of prodromal infection, clinical manifestations (mental or behavioral abnormalities, seizures, cognitive impairment, disturbance of consciousness, dyskinesia, central hypoventilation), complications (infection, electrolyte disturbance, liver or renal function injury, hypoproteinemia, stress ulcer, myocardial injury or arrhythmia), presence of tumor, laboratory indicators (antibody titers in CSF and blood, CSF leucocyte count, CSF protein levels, CSF-IgG levels, and the presence of other antibodies), magnetic resonance imaging (MRI) findings (unilateral lesions, bilateral lesions, or no lesions), disease severity [measured by Glasgow Coma Scale (GCS) score at admission, automatic discharge, and length of hospital stay], treatment regimens (first-line monotherapy, multiple first-line treatments, or second-line immunotherapy) and outcomes (improved, unhealed or deteriorated).

### 2.3 Statistical analysis method

For variables that follow a normal distribution, this study uses the mean ± standard deviation (*x̄ ± s*) to describe their centralized trend and discrete trend, and uses t-test to analyze the differences between the two groups. For variables that do not follow a normal distribution, this study uses the median and quartile range [*Median (Quartile Range)*] to describe their centralized trend and discrete trend, and uses nonparametric test to analyze the differences between the two groups. For categorical variables, this study uses the number of cases (constituent ratio) [*n (%)*] to describe, and uses chi-square test to analyze the differences between the two groups. *p* < 0.05 is considered statistically significant.

## 3 Results

### 3.1 Included samples

A total of 43 patients (*n* = 43) with anti-NMDAR encephalitis were included in this study, consisting of 20 male patients (*n* = 20, 46.51%) and 23 female patients (*n* = 23, 53.49%). A detailed comparison of the two groups is presented in Table 1.

**Table 1:** Comparison between two groups.

Variable	Male group (*n* = 20)	Female group (*n* = 23)	Statistics	*P*
Age [year, median (quartile range)]	37.00 (33.25, 54.50)	33.00 (23.00, 55.00)	−0.963	0.336^a^
Prodromal infection [*n* (%)]	10 (50.00)	9 (39.13)	0.467	0.494^b^
Secondary to viral encephalitis [*n* (%)]	3 (15.00)	1 (4.35)	0.453	0.501^b^
Clinical manifestations [*n* (%)]				
Mental or behavioral abnormalities	10 (50.00)	17 (73.91)	2.618	0.106^b^
Seizures	9 (45.00)	11 (47.83)	0.034	0.853^b^
Cognitive impairment	4 (20.00)	10 (43.48)	2.686	0.101^b^
Disturbance of consciousness	3 (15.00)	10 (43.48)	4.113	0.043^b^
Dyskinesia	2 (10.00)	5 (21.74)	0.392	0.531^b^
Central hypoventilation	1 (5.00)	5 (21.74)	1.297	0.255^b^
Complications [*n* (%)]	11 (55.00)	15 (65.22)	0.467	0.494^b^
Infection	6 (30.00)	12 (52.17)	2.161	0.142^b^
Electrolyte disturbance	6 (30.00)	8 (34.72)	0.111	0.739^b^
Liver or renal function injury	5 (25.00)	9 (39.13)	0.973	0.324^b^
Hypoproteinemia	4 (20.00)	9 (39.13)	1.856	0.173^b^
Stress ulcer	3 (15.00)	7 (30.43)	0.694	0.405^b^
Myocardial injury or arrhythmia	0 (0.00)	5 (21.74)	3.032	0.082^b^
Presence of concurrent tumor [*n* (%)]	1 (5.00)	5 (21.74)	1.297	0.255^b^
Laboratory indicators				
CSF antibody titers [median (quartile range)]	1:1 (1:1, 1:10)	1:10 (1:3.2, 1:32)	−2.870	0.004^a^
Negative serum antibody [*n* (%)]	5 (25.00)	1 (4.35)	2.275	0.131^b^
CSF leucocyte count [×10^6^/L, median (quartile range)]	34.00 (6.50, 117.50)	10.00 (6.00, 34.00)	−1.224	0.221^a^
CSF protein level [g/L, median (quartile range)]	0.6000 (0.4475, 0.8675)	0.3800 (0.3200, 0.5500)	−2.591	0.019^a^
CSF-IgG level [mg/L, median (quartile range)]	50.6500 (37.4500, 86.3750)	45.9000 (29.4000, 67.4000)	−0.548	0.584^a^
Overlay anti-MOG antibody	6 (30.00)	0 (0.00)	5.715	0.017^b^
MRI performance [*n* (%)]			−0.630	0.546^a^
Unilateral lesions	9 (45.00)	5 (21.74)	2.636	0.104^b^
Bilateral lesions	7 (35.00)	8 (34.72)	0.000	0.998^b^
No lesions	4 (20.00)	10 (43.48)	2.686	0.101^b^
Disease severity				
GCS score at admission [median (quartile range)]	15.00 (12.25, 15.00)	14.00 (10.00, 15.00)	−1.520	0.129^a^
Automatic discharge [*n* (%)]	1 (5.00)	8 (34.78)	4.075	0.044^b^
Length of hospital stay (day, *x̄ ± s*)	16.11 ± 8.399 (*n* = 19)	23.40 ± 16.093 (*n* = 15)	−1.593	0.127^c^
Treatment [*n* (%)]				
First-line monotherapy	8 (42.11) (*n* = 19)	5 (21.74) (*n* = 23)	2.019	0.155^b^
Multiple first-line treatments	11 (57.89) (*n* = 19)	18 (78.26) (*n* = 23)	2.019	0.155^b^
Second-line immunotherapy	3 (15.79) (*n* = 19)	6 (26.09) (*n* = 23)	0.186	0.666^b^
Outcomes [*n* (%)]				
Improved	18 (90.00)	14 (60.87%)	4.768	0.029^b^
Unhealed or deteriorated	2 (10.00)	9 (39.13)	4.768	0.029^b^

Statistics: a: nonparametric test; b: chi-square test; c: t-test. CSF, cerebrospinal fluid; IgG, glycoprotein G; MRI, Magnetic Resonance Imaging; GCS, Glasgow Coma Scale.

### 3.2 Statistical results

#### 3.2.1 Age

The median age of male group was 37.00 (33.25, 54.50) years old, while the median age of the female group was 33.00 (23.00, 55.00) years old. No significant difference was observed between the two groups (*Z* = −0.963, *p* = 0.336).

#### 3.2.2 Prodromal infection

In the male group, 10 cases (50.00%) had a history of prodromal infection, with 3 cases definitely diagnosed as viral encephalitis (15.00%); in the female group, 9 cases (39.13%) had a history of prodromal infection, including 1 case definitely diagnosed as viral encephalitis (4.35%). There were no significant difference between the two groups regarding the history of prodromal infection (*χ^2^* = 0.467, *p* = 0.494) or secondary to viral encephalitis (*χ^2^* = 0.453, *p* = 0.501).

#### 3.2.3 Clinical manifestations

##### 3.2.3.1 Mental or behavioral abnormalities

In the male group, 10 cases (50.00%) exhibited mental or behavioral abnormalities, compared to 17 cases (73.91%) in the female group. The difference was not statistically significant (*χ^2^* = 2.618, *p* = 0.106).

##### 3.2.3.2 Seizures

Seizures were reported in 9 cases (45.00%) in the male group and 11 cases (47.83%) in the female group, with no statistically significant difference observed (*χ^2^* = 0.034, *p* = 0.853).

##### 3.2.3.3 Cognitive impairment

Cognitive impairment was present in 4 cases (20.00%) of the male group and 10 cases (43.48%) of the female group, with no statistically significant difference (*χ^2^* = 2.686, *p* = 0.101).

##### 3.2.3.4 Disturbance of consciousness

Disturbance of consciousness occurred in 3 cases (15.00%) in male group and 10 cases (43.48%) in female group. This difference was statistically significant (*χ^2^* = 4.113, *p* = 0.043).

##### 3.2.3.5 Dyskinesia

Dyskinesia was observed in 2 cases (10.00%) of the male group and 5 cases (21.74%) of the female group, with no statistically significant difference (*χ^2^* = 0.392, *p* = 0.531).

##### 3.2.3.6 Central hypoventilation

Central hypoventilation was seen in 1 male patient (5.00%) and 5 female patients (21.74%), with no statistically significant difference (*χ^2^* = 1.297, *p* = 0.255).

#### 3.2.4 Complications

In the male group, there were 11 cases of complications (55.00%) compared to 15 cases in the female group (65.22%), with no statistically significant difference (*χ^2^* = 0.467, *p* = 0.494).

##### 3.2.4.1 Infection (including pneumonia or urinary tract infection)

Infections were reported in 6 cases (30.00%) in the male group and 12 cases (52.17%) in the female group; the difference was not statistically significant (*χ^2^* = 2.161, *p* = 0.142).

##### 3.2.4.2 Electrolyte disturbance

Electrolyte disturbances occurred in 6 cases (30.00%) in the male group and 8 cases (34.72%) in the female group, with no statistically significant difference (*χ^2^* = 0.111, *p* = 0.739).

##### 3.2.4.3 Liver or renal function injury

Liver or renal function injuries were noted in 5 cases (25.00%) in the male group and 9 cases (39.13%) in the female group, with no statistically significant difference (*χ^2^* = 0.973, *p* = 0.324).

##### 3.2.4.4 Hypoproteinemia

Hypoproteinemia was observed in 4 cases (20.00%) in the male group and 9 cases (39.13%) in the female group, with no statistically significant difference (*χ^2^* = 1.856, *p* = 0.173).

##### 3.2.4.5 Stress ulcer

A total of 3 cases (15.00%) in the male group and 7 cases (30.43%) in the female group had stress ulcers, with no statistically significant difference (*χ^2^* = 0.694, *p* = 0.405).

##### 3.2.4.6 Myocardial injury or arrhythmia

No case (0.00%) of myocardial injury or arrhythmia was reported in the male group, while 5 cases (21.74%) were reported in the female group, with the difference not statistically significant (*χ^2^* = 3.032, *p* = 0.082).

#### 3.2.5 Presence of concurrent tumor

1 male patient (5.00%, with lung cancer) and 5 female patients (21.74%, including 4 with ovarian teratomas and 1 with uterine tumor) were found to have concurrent tumors. No significant difference was observed between the two groups (*χ*^2^ = 1.297, *p* = 0.255).

#### 3.2.6 Laboratory indicators

CSF antibody titers in the male group were 1:1 (1:1, 1:10), while in the female group, they were 1:10 (1:3.2, 1:32). Female patients exhibited significantly higher antibody titers in CSF than male patients (*Z* = −2.870, *p* = 0.004). Regarding serum antibody negativity, 5 cases (25.00%) were observed in the male group compared to only 1 case (4.35%) in the female group. However, this difference was not statistically significant (*χ^2^* = 2.275, *p* = 0.131).

The leucocyte count in CSF of the male group was 34.00 (6.50, 117.50) × 10^6^/L, while in the female group, it was 10.00 (6.00, 34.00) × 10^6^/L. There was no significant difference between the two groups (*Z* = −1.224*, p* = 0.221). The CSF protein level in the male group was 0.6000 (0.4475, 0.8675) g/L, compared to 0.3800 (0.3200, 0.5500) g/L in the female group, which was significantly higher in males (*Z* = −2.591, *p* = 0.019). The CSF-IgG level in the male group was 50.6500 (37.4500, 86.3750) mg/L, while in the female group, it was 45.9000 (29.4000, 67.4000) mg/L, with no statistically significant difference observed (*Z* = −0.548, *p* = 0.584).

Anti-myelin oligodendrocyte glycoprotein (Anti-MOG) antibodies were detected in the CSF and serum of 6 patients in the male group (30.00%). In the female group, one patient tested positive for anti-*α*-amino-3-hydroxy-5-methyl-4-isoxazolepropionic acid 2 receptor (AMPA2R) antibodies in her CSF (4.35%), another patient had anti-Yo antibodies detected in her serum (4.35%), and a third patient had both glycine receptor 1 (GLyR1) and glial fibrillary acidic protein (GFAP) antibodies identified in her CSF (4.35%). Males were found to be more prone to the presence of superimposing anti-MOG antibodies compared to females (*χ^2^* = 5.715, *p* = 0.017).

#### 3.2.7 MRI findings

In the male group, there were 9 cases (45.00%) with unilateral lesions, while in the female group, there were 5 cases (21.74%). The difference between the two groups was not statistically significant (*χ^2^* = 2.636, *p* = 0.104). Additionally, there were 7 cases (35.00%) with bilateral lesions in the male group and 8 cases (34.72%) in the female group, with no statistically significant difference observed (*χ^2^* = 0.000, *p* = 0.988). Furthermore, 4 males (20.00%) and 10 females (43.48%) had no MRI lesions, and the difference was also not statistically significant (*χ^2^* = 2.686, *p* = 0.101).

#### 3.2.8 Disease severity

The GCS score at admission for the male group was 15.00 (12.25, 15.00), while the female group had a GCS score of 14.00 (10.00, 15.00). There was no significant difference between the two groups (*Z* = −1.520, *p* = 0.129). 1 patient (5.00%, refused treatment) in the male group and 8 patients (34.78%, 3 cases did not recover, and 5 cases deteriorated) in the female group discharged automatically. Female patients were more likely to be discharged automatically (*χ^2^* = 4.075, *p* = 0.044). The average duration of hospitalization was 16.11 ± 8.399 days for the male group (*n* = 19) and 23.40 ± 16.093 days for the female group (*n* = 15). No significant difference was found in the length of hospital stay between the two groups (*t* = −1.593, *p* = 0.127).

#### 3.2.9 Treatment

In the male group (1 patient discharged without treatment, *n* = 19), 8 cases (42.11%) received first-line monotherapy with methylprednisolone, while the remaining 11 cases (57.89%) required multiple first-line treatments, including methylprednisolone, intravenous immunoglobulin (IVIG), and plasma exchange (PE). In the female group (*n* = 23), 5 cases (21.74%) received first-line monotherapy with methylprednisolone, whereas the remaining 18 cases (78.26%) required multiple first-line treatments. There was no significant difference between the two groups (*χ^2^* = 2.019, *p* = 0.155).

In the male group, 3 patients (15.79%, all using rituximab) required initiation of second-line treatment, while in the female group, 6 patients (26.09%, including 5 using rituximab, 1 using ofatumumab) needed to start second-line immunotherapy. There was no significant difference between the two groups (*χ^2^* = 0.186, *p* = 0.666).

#### 3.2.10 Outcomes

Based on the improvement of Modified Rankin Scale (mRS) at discharge, patients were classified into two categories: improved (reduction of mRS score by ≥1), unhealed or deteriorated (reduction of mRS score by <1 or increase in mRS score). In the male group, 18 cases (90.00%) showed improved, which was higher than the 14 cases (60.87%) in the female group. In the male group, there were 2 patients (10.00%, including 1 patient who refused treatment and left the hospital automatically) who were unhealed or deteriorated, and there were 9 patients (39.13%, including 8 patients who left the hospital automatically) in the female group. The difference between the two groups was statistically significant (*Z* = 4.768, *p* = 0.029).

### 3.3 Summary

Female patients were more likely to have a decreased level of consciousness compared to male patients (*χ^2^ =* 4.113, *p* = 0.043), and the antibody titer in CSF was also higher in females than in males (*Z* = −2.870, *p* = 0.004). Conversely, the CSF protein level was higher in male patients (*Z* = −2.591, *p* = 0.019), and they were more likely to have superimposing anti-MOG antibodies (*χ^2^* = 5.715, *p* = 0.017). Furthermore, the improvement rate following treatment was lower for female patients than for male patients (*Z* = 4.768, *p* = 0.029), and the family members of female patients were more likely to choose automatic discharge (*χ^2^* = 4.075, *p* = 0.044).

## 4 Discussion

Through the study, we found that sex differences generally exist in patients with anti-NMDAR encephalitis, encompassing clinical manifestations, laboratory indicators and disease outcomes. Previous reports on sex differences in anti-NMDAR encephalitis have been limited. One study conducted in Southeast Asia suggested that clinical characteristics may differ between sexes (Tan et al., 2023). The authors noted that male were more prone to early seizures and insomnia, while and females were four times likelier than males to develop movement disorders or have underlying neoplasms. Furthermore, some scholars have indicated that female patients tend to present with more clinical symptoms than male patients, and the peak-stage background activity (BA) in electroencephalogram (EEG) was worse in female patients than in male patients (Miao et al., 2019).

In this study, the GCS score of female patients at admission was lower than that of male patients, although the difference was not statistically significant. However, as the disease progressed, the likelihood of a decline in consciousness was higher in the female group compared to the male group. This indicates that female patients are more prone to progress and worsen, resulting in a decline in the level of consciousness.

Animal experiments have demonstrated that the expression of NMDAR in the preoptic area of male rats is higher than that of female rats (Hsu et al., 1999). This finding may also be applicable to other areas of the brain or across different species. The elevated expression of NMDAR may help mitigate extensive neurological damage during the onset of anti-NMDAR encephalitis. Research has shown that female patients with high antibody titers (HAT) in CSF exhibit greater reductions in cerebral blood flow (CBF) in regions such as the left post cingulum gyrus, left precuneus, left calcarine, and left middle cingulum gyrus compared to male patients with the same antibody titers (AT) (Miao and Wang, 2024). The decrease of CBF indicates more pronounced brain tissue inflammation and more severe function damage. These dominant hemispheric limbic systems are primarily associated with emotion, cognition and consciousness, which may explain why female patients experience more severe symptoms and are more prone to consciousness disorders.

A study involving 312 patients with AE, including 197 patients with anti-NMDAR encephalitis (accounting for 63.1%), found that disturbances in consciousness were linked to elevated specific antibody titers (Shan et al., 2024). In our study, the antibody titers in the CSF of female patients were significantly higher than those in male patients, which may contribute to a more pronounced decline in the level of consciousness among female patients compared to their male counterparts.

Not only are there differences in antibody titers, but the probability of serum antibody negativity in male patients was also higher than that in female patients (25.00% vs. 4.35%). This raises the question: why do female patients exhibit higher antibody titers? AE is often associated with cross-immune responses triggered by infections or tumors. Anti-NMDAR encephalitis has a particularly close relationship with tumors, especially ovarian teratomas. This study found that the prevalence of tumors in female patients was significantly higher than in male patients (21.74% vs. 5.00%). This difference may contribute to the elevated CSF antibody titers observed in female patients.

Opinions differ regarding the relationship between antibody titers and disease severity. Cai et al. (2021) suggested that patients with high antibody titers are more prone to mental symptoms, which they argue are not necessarily linked to disease severity. However, more evidences point to a correlation between antibody titers and the severity and prognosis of anti-NMDAR encephalitis. Miao et al. (2019) found that patients with high CSF antibody titers exhibited more clinical symptoms compared to those with low titers. Similarly, Zhang et al. (2018) reported that the proportion of patients with strongly positive anti-NMDAR antibodies in the severe group (48.7%) was higher than that in the non-severe group (29.2%). A study involving 250 patients with anti-NMDAR encephalitis indicated that both the CSF and serum antibody titers of patients with poor prognoses or teratomas were higher than those of patients with good prognoses or no tumors (Gresa-Arribas et al., 2014). This aligns with the findings for female patients in our study. Furthermore, improvements in symptoms have been linked to decreases in antibody titers (Dalmau et al., 2011). Patients with persistent anti-NMDAR antibodies in their CSF are at greater risk for subsequent recurrence and poor long-term prognoses (Ciano-Petersen et al., 2023).

We also believe that antibody titer is closely related to the onset and progression of the disease. In this study, the incidence of consciousness disorders in female patients was significantly higher than that in male patients, with the incidence of central hypoventilation being four times greater in females. Although not statistically significant, the rates of other clinical symptoms and complications were generally higher in female patients compared to male patients.

Although the antibody titer in male patients was lower than that of female patients, the CSF protein level was higher in males. This discrepancy may be linked to physiological factors, as men typically exhibit high CSF protein content (Candeloro et al., 2024). An analysis of 6,068 CSF samples by McCudden et al. (2017) concluded that males exhibited significantly higher levels of CSF total protein (CSF-TP) compared to females across all age groups. Specifically, the CSF protein level in males was found to be, on average, 10.8 mg/dL higher than that in females (Fautsch et al., 2023). These differences might be associated with endocrine variations between the sexes. Additionally, disparities in height and Body Mass Index (BMI) could contribute to a larger CSF volume and blood/brain interfaces in men (McCudden et al., 2017; Fautsch et al., 2023).

The research results also indicated that male patients with anti-NMDAR encephalitis were more frequently had overlapping anti-MOG antibodies (30.00% vs. 0.00%). Previous literature has noted that this patient population predominantly consists of young males (Ding et al., 2021). A study involving 49 cases of MOG antibody and NMDAR antibody overlap syndrome (MNOS) reported that all patients were young males (Du et al., 2024). Additionally, a non-randomized controlled study of 17 patients with anti-NMDAR encephalitis and coexisting anti-MOG antibodies found a male-to-female ratio of 14:3 (Ding et al., 2025). This phenomenon may be linked to the fact that male patients are more likely to experience dysfunction of the blood-cerebrospinal fluid barrier (BCSFB) compared to female (44% vs. 20.1%) (Candeloro et al., 2024), which can result in increased exposure to multiple intracranial antigens including MOG and NMDAR.

Even though male patients with anti-NMDAR encephalitis have higher levels of CSF protein and are more frequently have overlapping anti-MOG antibodies, these factors do not appear to influence their condition or outcomes. In contrast, a significant majority of female patients in this study required two or more first-line immunotherapyies, with approximately one-quarter needing to initiate second-line immunotherapy. Despite these intensive treatments, the proportion of female patients with good outcomes in acute phase is lower than that of male patients, and their families were significantly more likely to choose voluntary discharge, although most indicate that they will continue treatment at lower-level hospitals.

The outcome of anti-NMDAR encephalitis is influenced by numerous factors. According to literature reports, disorders of consciousness are considered a risk factor for poor prognosis in these patients (Gong et al., 2021; Mo et al., 2020). The prognosis of anti-NMDAR encephalitis associated with malignant tumors may depend on the prognosis of the tumor itself (Hara et al., 2012). However, patients with tumors often fail to receive tumor resection during the acute phase of encephalitis, and many patients’ tumors may remain in a dormant state. Additionally, as previously mentioned, high antibody titers are linked to poor prognosis. Compared to male patients, female patients experience more severe consciousness disorders, are more likely to have tumors, especially ovarian teratomas, and exhibit higher antibody titers. These factors may contribute to a significantly lower rate of improvement during the acute phase for female patients compared to males.

There are few reports in the literature addressing prognostic differences influenced by sex. Wang (2020) suggests that the one-year recovery rate for female patients may be higher than that for male patients, which contrasts with the findings of our study conducted during the acute phase. It is important to note that most of the male patient cases referenced in the article were derived from literature case reports and excluded those without tumor. This raises questions about the representativeness of the enrolled patients. Nonetheless, the impact of sex differences on long-term prognosis remains a significant area for future research.

## 5 Conclusion

Through our research, we have found that female patients with anti-NMDAR encephalitis face greater challenges and difficulties than males. Although male patients exhibit higher CSF protein levels and are more frequently have overlapping anti-MOG antibodies, female patients present higher CSF antibody titers and are more prone to decreased levels of consciousness. This complicates treatment of female patients, who often require multiple first-line immunotherapy treatments and may even need to initiate second-line immunotherapy. Despite these efforts, the improvement rate among female patients is significantly lower than that of male patients. We speculate these factors lead families of female patients to be more inclined to automatic discharge.

The clinical differences attributed to these gender factors may be linked to variations in pathophysiological mechanisms between the sexes, such as tumor incidence (Tan et al., 2023), expression of NMDAR (Hsu et al., 1999), reductions in CBF (Miao and Wang, 2024), and BCSFB vulnerability (Candeloro et al., 2024), as previously mentioned. Furthermore, we speculate whether hormone levels (Rehan et al., 2024), cultural or economic factors may also play a role, warranting further investigation.

Considering the potential poor outcomes of female patients during the acute phase, we may be more proactive in selecting treatment plans for female patients compared to male patients to help reduce the risk of adverse outcomes. Especially IVIG and rituximab may be beneficial for the long-term prognosis of female patients (Wang, 2020).

## 6 Deficiency and inspiration

Anti-NMDAR encephalitis is a relatively rare autoimmune disease of the central nervous system (CNS), which has resulted in a limited number of cases in this study, potentially affecting the reliability of the statistical results and leading to type I or type II statistical errors. Therefore, continued case collection of cases in the future is essential. At the same time, this study primarily focuses on sex differences during the early stages of the disease. Although female patients may experience a poorer early recovery compared to male patients, this finding lacks reference significance for long-term prognosis and deserves further research.

## Data Availability

The raw data supporting the conclusions of this article will be made available by the authors, without undue reservation.
